# Supplementary material for: Opposing Actions of Octopamine and Tyramine on Honeybee Vision
Source: Biomolecules. 2021 Sep 17;11(9):1374. doi: 10.3390/biom11091374 (PMC8468255; doi:10.3390/biom11091374)
Supplement: Supplementary file 1 [file biomolecules-11-01374-s001.zip › biomolecules-1369591-supplementary.pdf]

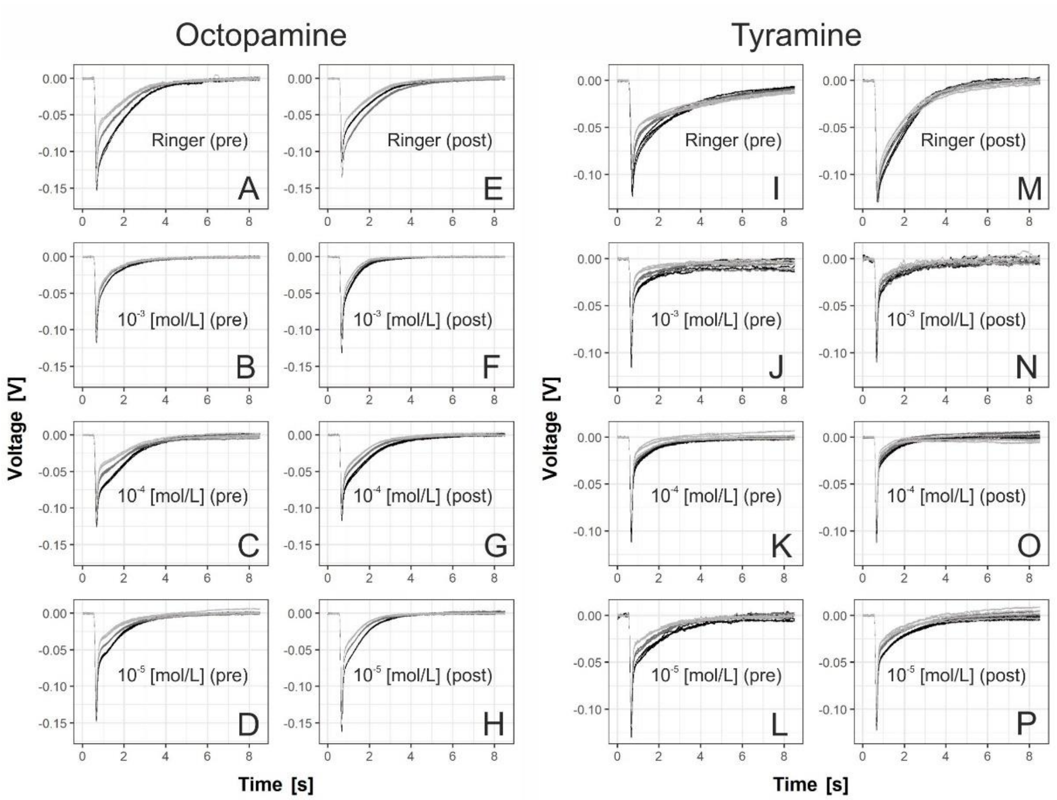

Figure S1. Raw data of eight example bees for the ERG experiments. Different colours show the different light intensities (light grey−36%, grey−59%, black−100%) and each pair of pre and post graphs represents one individual. (**A**−**H**): Receptor activity in response to four trials of each light intensity before (pre; A−D) and after (post; E−H) octopamine application. The upper 2 panels (A and E) illustrate the Ringer control. (**I**−**P**): Same as A−H but for tyramine application. Note: the four repetitions of each intensity induce almost identical activity, illustrating our reliable recording and stimulus application.

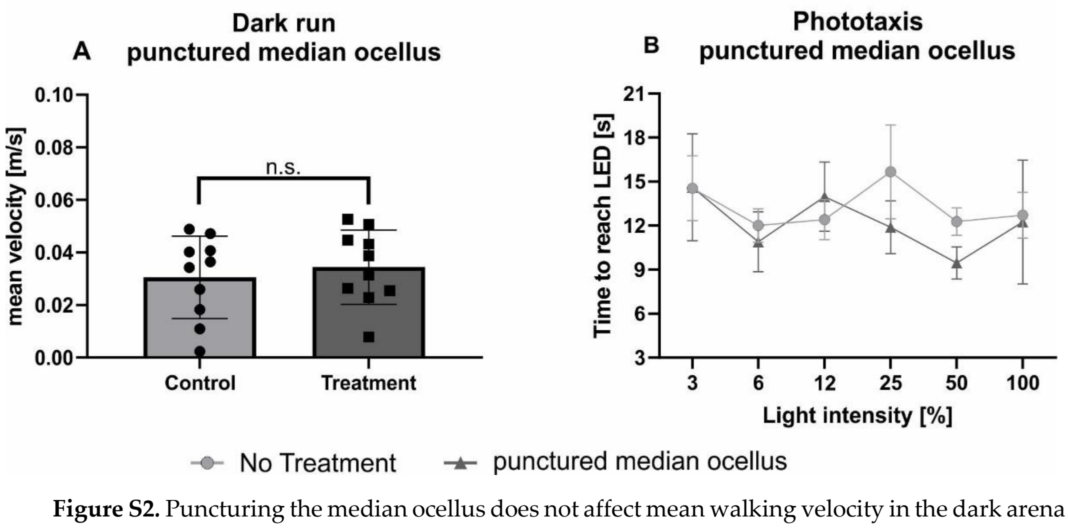

Figure S2. Puncturing the median ocellus does not affect mean walking velocity in the dark arena or walking time towards the switched-on LEDs. Control bees are shown in black. Honeybees with a punctured median ocellus are shown in grey. A: Average velocity (mean + standard deviation) of honeybees during one minute of constant movement in the dark arena. Groups did not differ significantly in their walking velocity in the dark ($T = 0.58$, $n_{tyramine} = 10$, $n_{control} = 10$; $p = 0.57$). B: Average walking time (mean + standard error) towards the different light sources. Light intensity did not affect walking time (ANOVA with RM, factor light intensity: $F_{(5, 90)} = 1.4$, $p = 0.25$). Puncturing the median ocellus also did not affect walking times (ANOVA, factor treatment: $F_{(1, 18)} = 0.2$, $p = 0.67$).
